# The panniculus carnosus muscle: a missing link in the chronicity of heel pressure ulcers?

**DOI:** 10.1098/rsif.2021.0631

**Published:** 2022-02-23

**Authors:** N. Jannah M. Nasir, Alberto Corrias, Hans Heemskerk, Eng Tat Ang, Julia H. Jenkins, S. J. Sebastin, Lisa Tucker-Kellogg

**Affiliations:** ^1^ Cancer and Stem Cell Biology and Centre for Computational Biology, Duke-NUS Medical School, Singapore; ^2^ Department of Biomedical Engineering, National University of Singapore; ^3^ Department of Anatomy, Yong Loo Lin School of Medicine, National University of Singapore, Singapore; ^4^ Department of Hand and Reconstructive Microsurgery, National University Health System, Singapore

**Keywords:** chronic wounds, pressure injuries, functional anatomy of heel, lineage tracing in mouse, biomechanics of muscle, muscle regeneration

## Abstract

Chronic and recurring pressure ulcers (PUs) create an unmet need for predictive biomarkers. In this work, we examine the panniculus carnosus, a thin cutaneous muscle, traditionally considered vestigial in humans, and ask whether the panniculus may play a role in the chronicity and reinjury of heel PUs. To determine whether humans have a panniculus muscle layer at the heel, we dissected eight cadavers. To assess the influence of the panniculus layer on PU, we performed computational simulations of supine weight bearing. Finally, we assessed panniculus regeneration in fluorescent mice. Results show a panniculus layer present in all cadavers examined. Simulations show a thin layer of panniculus muscle causes a dramatic decrease in the volume of soft tissue experiencing high strain and stress, compared to a heel without a panniculus. Importantly, in the mouse model, the panniculus fails to regenerate after PU, even when other cutaneous layers had fully regenerated. Our work shows that the panniculus is able to redistribute load around the heel bone, which might allow it to prevent PUs. Moreover, it is highly susceptible to incomplete regeneration after PU. Poor panniculus regeneration after PU might be a predictive anatomical biomarker for recurrence, and this biomarker should be evaluated prospectively in future clinical trials.

## 1. Introduction

Pressure ulcers (PUs) are painful wounds that are slow to heal, laborious to prevent and costly to manage. For example, the UK spends £507.0 to £530.7 million per year on PU management [1]. PUs are regions of localized skin and/or tissue damage caused by pressure and/or shear [2], also known as decubitus ulcers, pressure injuries or bedsores. The prevalence rate of PUs in hospitalized US patients ranges from 2.7% to 29% [3–5]. Intriguingly, PUs are classified as chronic wounds, which raises the question of why they do not regenerate as well as acute wounds. The vast majority of PU patients are not diabetic [6], and some are young (i.e. not elderly, for example, patients with spinal cord injury [7]).

In addition to slow healing, PUs have a high rate of recurrence at the same site (27–63%, depending on the study [8–12]), and efforts to predict PU recurrence have not yet been effective [13–15]. Work is underway to identify criteria, other than closure of the epithelium, for judging the quality of wound healing [16]. There remains an unmet need to identify which superficially healed PUs are at elevated risk of PU recurrence.

Muscle tissue is moderately firm and elastic, so it creates an ideal interface between hard bone and soft tissue by absorbing and redistributing load [17,18]. Heel regions at highest risk of PU may appear to lack any muscle, but a report in 2009 described the presence of a layer of panniculus carnosus (panniculus) muscle in the subcutaneous tissue of the heel [19]. Unfortunately, this finding was not pursued beyond *n* = 3 cadavers. In humans, the panniculus is considered vestigial and has been largely ignored [20]. Humans do have a panniculus layer documented in specific anatomical regions such as the neck and palm, and many people have a layer of panniculus muscle in additional regions, such as over the ribs [21]. In other mammals, the panniculus enables skin twitching, thermoregulation, cutaneous wound contraction and defence against skin irritation [20,22]. There is no known function of the panniculus in humans, except in the palm where it has been proposed to cushion the wrist and to protect the nerves and vessels at the ulnar canal [23].

In this study, we ask whether a panniculus layer might play an underappreciated role in PU prevention, healing and recurrence. We start by asking what fraction of humans have a panniculus layer in the heel, because the heel is a pressure-vulnerable location and second most common site for PUs [24–26]. We next ask whether the panniculus would protect the heel from PUs. Finally, we study panniculus regeneration after a PU, looking for any clues that could explain or predict the increased risk of PU recurrence or reinjury after an initial injury.

## 2. Methods

### 2.1. Cadaver tissue preparation

Human heels were collected from cadaver lower limbs that were originally sourced from the United States for an orthopaedic dissection course. Specifically, the entire heel pad (including the non-weight bearing portion of the posterior heel) superficial to the calcaneum was surgically excised and fixed in 4% formaldehyde overnight at 4°C. After fixation, the tissues were divided and frozen prior to cryo-sectioning. Thirty-micrometre cross-sections were cut from the mid-portion of the frozen samples (skin, fascia and fat pad). Frozen sections were stained with haematoxylin and eosin (H&E). The remaining tissues were paraffin-embedded and sectioned into 10 micrometre cross-sections and stained with H&E and Masson's trichrome to observe the general morphology. We were not able to control all aspects of tissue breakdown and degeneration of these samples, particularly for high-resolution features, and we were unable to conclusively evaluate striation. All methods were carried out in accordance with the National Healthcare Group Domain Specific Review Board (reference no. 2017/00935).

### 2.2. Three-dimensional model of the heel

A simplified geometry of the heel was adopted in this study [27]. Two types of simulations were run. In the first, a 1.5 mm thick layer of representing the panniculus was inserted between the skin layer (2 mm thick) and the fatty soft-tissue layer (3 mm thick). In the second simulation, the panniculus layer was replaced by a layer of fatty soft tissue of the same thickness. In both simulations, the Achilles' tendon (5 mm thick) and a spheroidal calcaneus bone completed the geometry. A force was applied to the bottom of the heel in the upward direction to simulate the contact reaction force of the bed due to the weight of the foot. A value of F=12 N was chosen well within the range of reported weights of the foot [28]. A zero-displacement boundary condition was imposed to the top and side of the geometry.

A nearly incompressible hyperelastic neo-Hookean material law was used. The strain energy function (W) was divided in two components: a volumetric $\left(W_{\mathrm{vol}}\right)$ and an isochoric one $\left(W_{\mathrm{iso}}\right)$ [29,30],

$$W=W_{\mathrm{vol}}+W_{\mathrm{iso}}=\frac{K}{4}(I_{3}-1-2\ln\left(\sqrt{I_{3}}\right)+\frac{\mu }{2}(\bar{I_{c}}-3),$$

where $I_{3}=\det(C)$ is the third Cauchy–Green invariant, *C* is the right Cauchy–Green tensor, $\bar{I_{c}}$ is the first isochoric Cauchy–Green invariant, $\bar{I_{c}}=I_{3}^{-(1/3)}tr(C).\;\;\mu$ and *K* are the material parameters. μ is the shear modulus, μ=E/(2(1+ν)), where *E* is Young's modulus and ν is Poisson's ratio. *K* is the bulk modulus, K=μ/(1−2ν). _._ The values of μ and *K* are reported in electronic supplementary material, table S1 alongside the corresponding values of *E* and ν for all the tissues involved as well as the reference publication.

### 2.3. Mice

**Figure 1.**
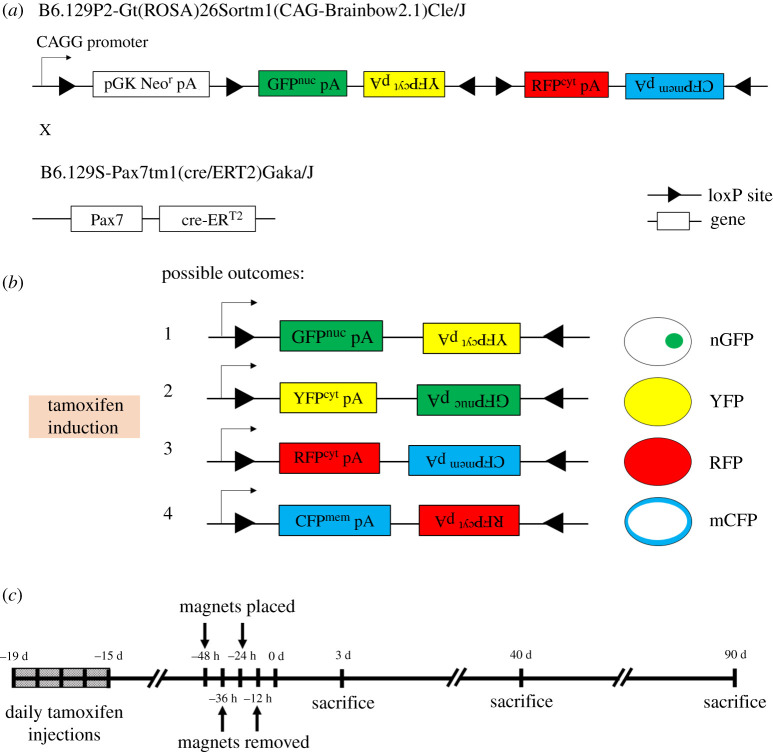
Set-up of the magnet-induced PU on the dorsal skinfold of mice. (*a*,*b*) Upon tamoxifen induction, the confetti transgene will be recombined specifically in Pax7 + cells, to express one of the following four proteins: GFP^nuc^ with a nuclear localization signal, cytoplasmic YFP^cyt^, cytoplasmic RFP^cyt^ or membrane-targeted (upon fusion, sarcolemma-targeted) CFP^mem^. Upside-down text indicates antisense genes not expressed. (*c*) Experimental schedule of PU induction and sacrifice for tissue harvest. Tamoxifen is injected two weeks prior to magnet placement.

Animal experiments were approved by the institutional animal care and use committee (IACUC SHS/2016/1257) of SingHealth, Singapore. To conditionally label Pax7 + muscle satellite stem cells in mice, the Pax7-Cre-ERT2 mouse was crossed with the Brainbow2.1 (confetti) mouse. Pax7-Cre-ERT2 provides the Cre-ERT2 transgene downstream of the Pax7 stop codon, thereby limiting the Cre-ERT2 expression to Pax7 + cells. The Cre-ERT2 system provides tamoxifen-dependent induction of Cre recombinase, so that affected cells carry heritable rather than transient modification. Upon tamoxifen treatment, the Cre induction causes recombination of the confetti construct at its loxP loci, leading to gene expression of one of the four fluorescent proteins in the construct. The fluorescent proteins are mCerulean (CFP^mem^), hrGFP II (GFP^nuc^), mYFP (YFP^cyt^) and tdimer2(12) (RFP^cyt^). CFP^mem^ contains a localization sequence enabling its transport to the myofibre membrane (sarcolemma), while GFP^nuc^ contains a nuclear localization sequence. YFP^cyt^ and RFP^cyt^ have no localization sequences and are expected to localize to the cytoplasm. Figure 1*a*,*b* illustrates the transgenic mouse model.

### 2.4. Murine injury model

Mice were shaved and fur removed by hair removal cream (Veet, Singapore) prior to injury. Muscle PUs were created in 4 to 5-month-old transgenic mice by applying a pair of ceramic magnets (Magnetic Source, Castle Rock, CO, part number: CD14C, grade 8) to the dorsal skinfold, which includes skin, adipose tissue, panniculus muscle and loose areolar tissue. The magnets were 5 mm thick and 12 mm in diameter, with an average weight of 2.7 g and pulling force of 640 g. To minimize the effect of hair follicle (HF) cycling, magnets were placed on ‘white' skin i.e. on skin where HFs are in telogen or resting phase. PU induction is performed in two cycles. Each cycle is made up of a 12-h period of magnet placement followed by a 12-hour period of magnet removal. Time points post-injury are measured from the end of last magnet cycle (figure 1*c*). This procedure induces two pressure wounds on the back of the mouse, on the left and right side of the dorsal skinfold. Prior studies have shown that unlike other types of mouse wound healing, the magnet-induced ulcer remains open, rather than closing by contraction [31–33]. The mice were given an analgesic (buprenorphine at 3.33 µl g^−1^) prior to magnet placement and again prior to magnet removal. The mice were individually housed and free to move. Food and water were given ad libitum. Figure 1*c* illustrates the experimental set-up of the murine injury model.

## 3. Results

### 3.1. The panniculus carnosus layer is present in the human heel

**Figure 2.**
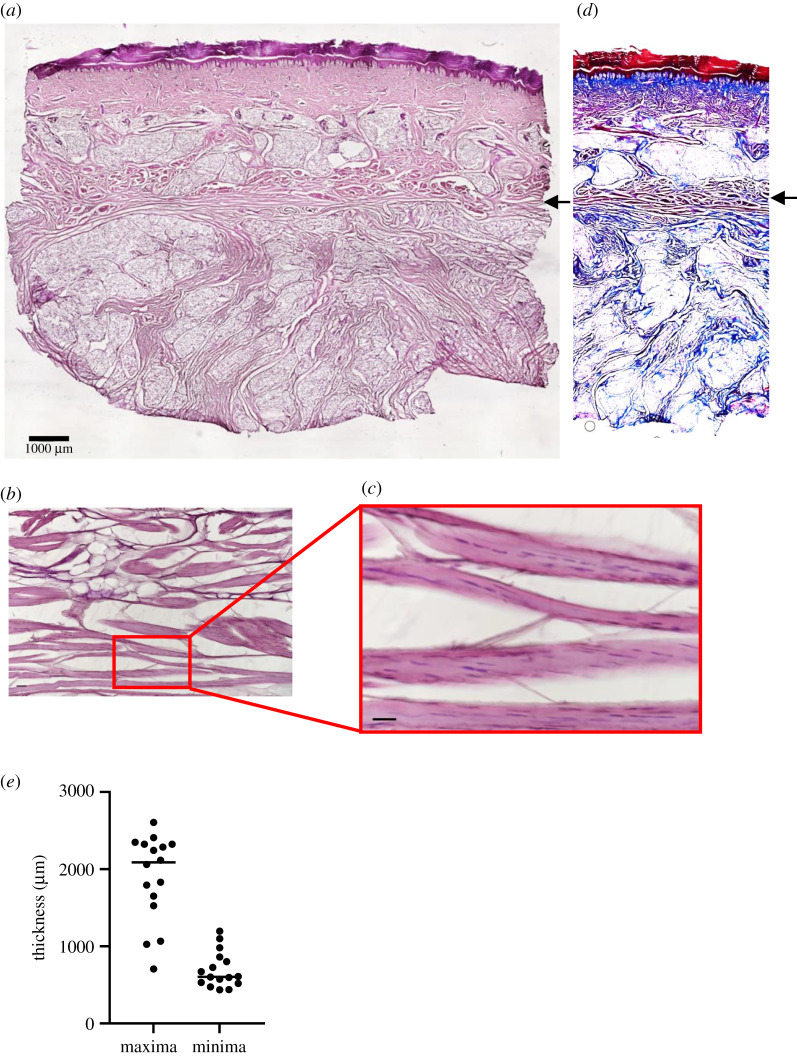
A layer of panniculus is found in heel tissues of human cadavers. (*a*) A cross-section of human cadaveric heel tissue stained with H&E. Panniculus carnosus is indicated by black arrow. Scale bar is 1000 µm. (*b*) A cross-section of human cadaveric heel tissue stained with Masson's trichrome reagent. Note the muscle fibres (black arrow) are stained red, while collagen is stained blue. (*c*,*d*) Close-ups of muscle fibres in the cadaveric heels. Scale bars are 50 µm. (*e*) Distribution of minimal and maximal thickness of panniculus layer in 16 cadaveric heel tissues (two samples from eight patients).

Eight heels from the right limbs of eight human cadavers (of mean age 80.25 ± 11.1 yr and from both sexes; electronic supplementary material, table S2) were dissected. Upon histological analysis, a layer of muscle was found between the dermis and fat pad in all of the heels (figure 2*a*–*c*). The muscle layer was distinct from the columnar layers of collagen (figure 2*d*) as shown by Masson's trichrome staining. Between individuals and across each section, the panniculus of the heel varied in thickness (electronic supplementary material, figure S1*a*,*b*), and the average of the maximal thicknesses was 1712 ± 546 µm, while the average minimal thickness was 630 ± 162 µm (figure 2*e*). There were no significant differences in panniculus thicknesses due to sex (electronic supplementary material, figure S1*c*,*d*) or age (electronic supplementary material, figure S1*e*,*f*). Immunofluorescence staining for alpha-smooth muscle actin was negative in the panniculus muscle fibres (electronic supplementary material, figure S2).

### 3.2. The panniculus improves load distribution in biomechanical simulations of the human heel

**Figure 3.**
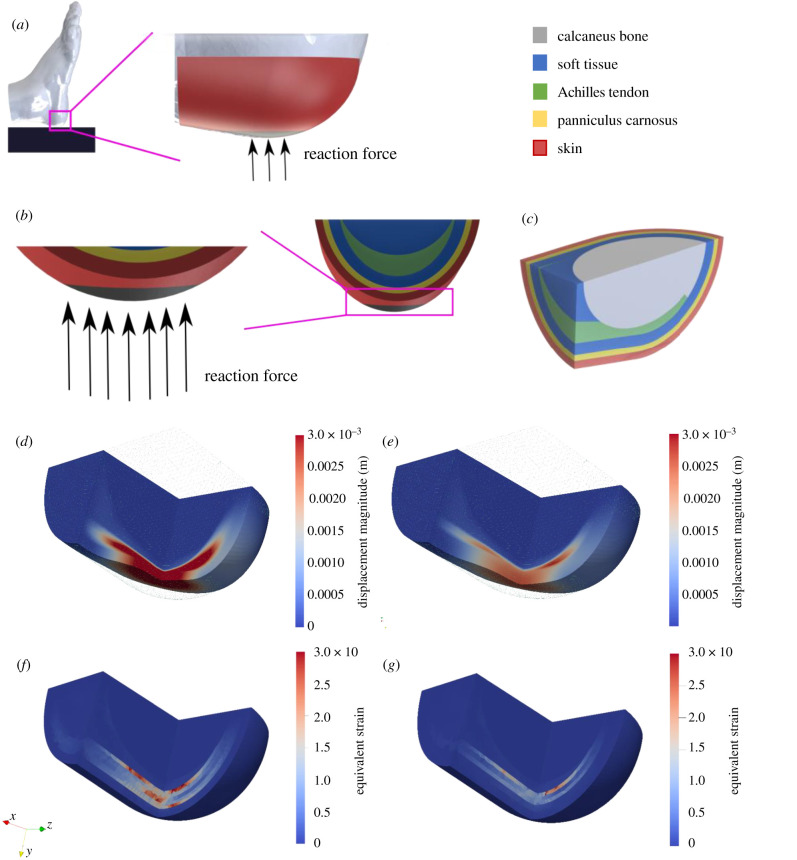
Simulation set-up and simulation results of the heel without a panniculus against results with a panniculus. (*a*) Schematic view of a foot lying on a solid support. In the simulations, the support is substituted by a reaction force due to the weight of the foot applied to our simplified geometry (in red, not to scale). (*b*) Detailed view of the bottom of the heel where the reaction force is applied (area in black). (*c*) Three-dimensional section view of the various tissue layers: skin is shown in red, panniculus in yellow, soft tissue in blue, Achilles's tendon in green and calcaneus bone in light grey. The mesh has been clipped in order to visualize internal stress and strains. (*d*) Shows displacement in a deformed geometry in the absence of the panniculus layer, whereas (*e*) shows displacement with panniculus present. The undeformed geometry is depicted as a green wireframe. (*f*) Shows equivalent strain shown in the undeformed geometry without a panniculus and (*g*) with a panniculus.

**Figure 4.**
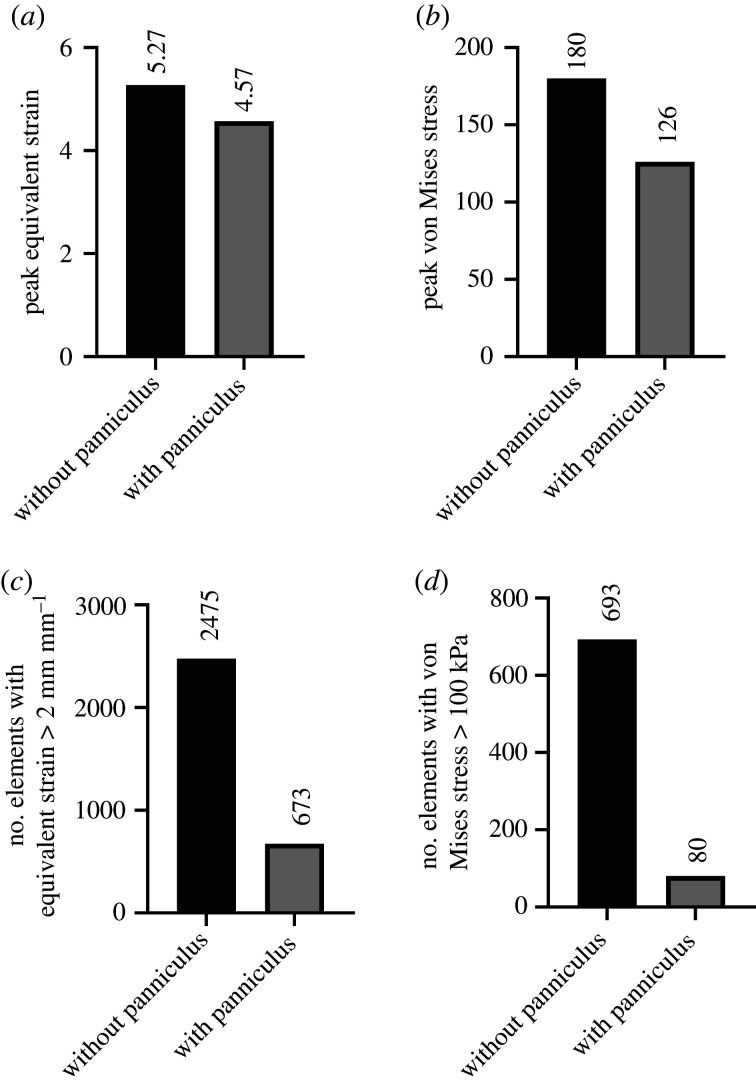
Simulation results of the heel without a panniculus against results with a panniculus. (*a*,*b*) Measures of (*a*) peak equivalent strain and (*b*) peak von Mises stress with the panniculus layer present compared to without the panniculus. (*c*,*d*) Comparison of the number of finite elements in the mesh with (*c*) equivalent strain above 2 and (*d*) von Mises stress above 100 kPa, for models with a panniculus versus without a panniculus. No error bars are shown for these calculations because our deterministic simulation provides only one value for each output.

Having observed its supposedly vestigial presence in human heels, we next sought to understand whether its presence mattered. To study how the panniculus could alter load distribution at the dorsal corner of the heel (analogous to a supine patient), we undertook a biomechanical simulation with finite-element modelling. We constructed a simplified three-dimensional model of tissue layers covering the calcaneus bone in a human heel (figure 3*a*–*c*) and applied an external force representing the weight of a resting foot. Figure 3*d*–*g* shows a section of the heel, coloured for the levels of displacement and equivalent strain that occurred when the panniculus was present or absent. The presence of the panniculus caused a decrease in overall displacement (figure 3*d*,*e*; electronic supplementary material, figure S3*a*,*b*), 13% decrease in peak equivalent strain (4.57 with panniculus versus 5.27 without; figures 3*f*,*g* and 4*a*) and 30% decrease in peak von Mises stress (126 kPa with panniculus versus 180 kPa without; figure 4*b*; electronic supplementary material, figure S3*c*,*d*). Remarkably, the presence of a panniculus layer caused a 73% decrease in the number of elements in the mesh with equivalent strain above 2 mm mm^−1^ (figure 4*c*) and an 89% decrease in the number of elements in the mesh with von Mises stress above 100 kPa (figure 4*d*).

### 3.3. Murine model of pressure ulcer exhibits death of tissue layers

**Figure 5.**
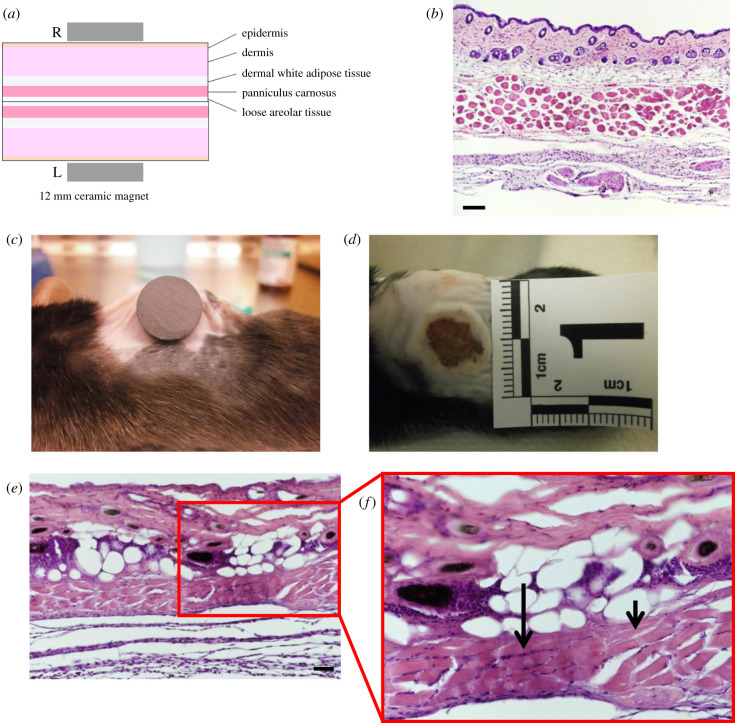
Murine injury model causes cell death. (*a*) Schematic of the layers present in the dorsal skinfold. (*b*) A H&E stained section of healthy murine dorsal skinfold, cut to show a cross-section of the muscle fibres. Scale bar is 50 µm. (*c*) Image of a 12 mm magnet on the dorsal skinfold of a C57BL6 mouse during PU induction. (*d*) A photograph of the external wound at 1 d after magnet-induced PU. (*e*) A cross-section (scale bar is 50 µm) and (*f*) close-up of the dorsal skinfold 3 d after magnet-induced PU. Small arrow indicates karyolysis and long arrow indicates karyorrhexis.

The dorsal skinfold of mice contains the following parallel layers: a thin epithelium (epidermis), a thicker layer of connective tissue (dermis), an adipose layer (dermal white adipose tissue), a layer of panniculus muscle and a thick layer of loose areolar tissue (figure 5*a*,*b*). One day following magnet-induced PU, an external surface wound can be observed on the skin (figure 5*c*,*d*). Wound tissues at 3 d post-injury showed characteristics of cell death, such as karyolysis, karyorrhexis and acidification (eosinification), as shown in the H&E staining of the panniculus (figure 5*e*,*f*). Secondary indications of injury include keratinocyte and adipocyte cell death. The diameter of the external wound contracted gradually from 9.44 ± 0.8 mm at day 1, to 7.79 ± 1.4 mm at day 7, to 4.45 ± 1.5 mm at day 14 and the time to full closure was 18–21 d. The diameter of the muscle defect was 7.82 ± 1.4 mm at day 3, 6.12 ± 0.7 mm at day 10 and 4.92 ± 0.7 at day 90.

### 3.4. Murine panniculus layer does not fully regenerate following magnet-induced pressure ulcer

**Figure 6.**
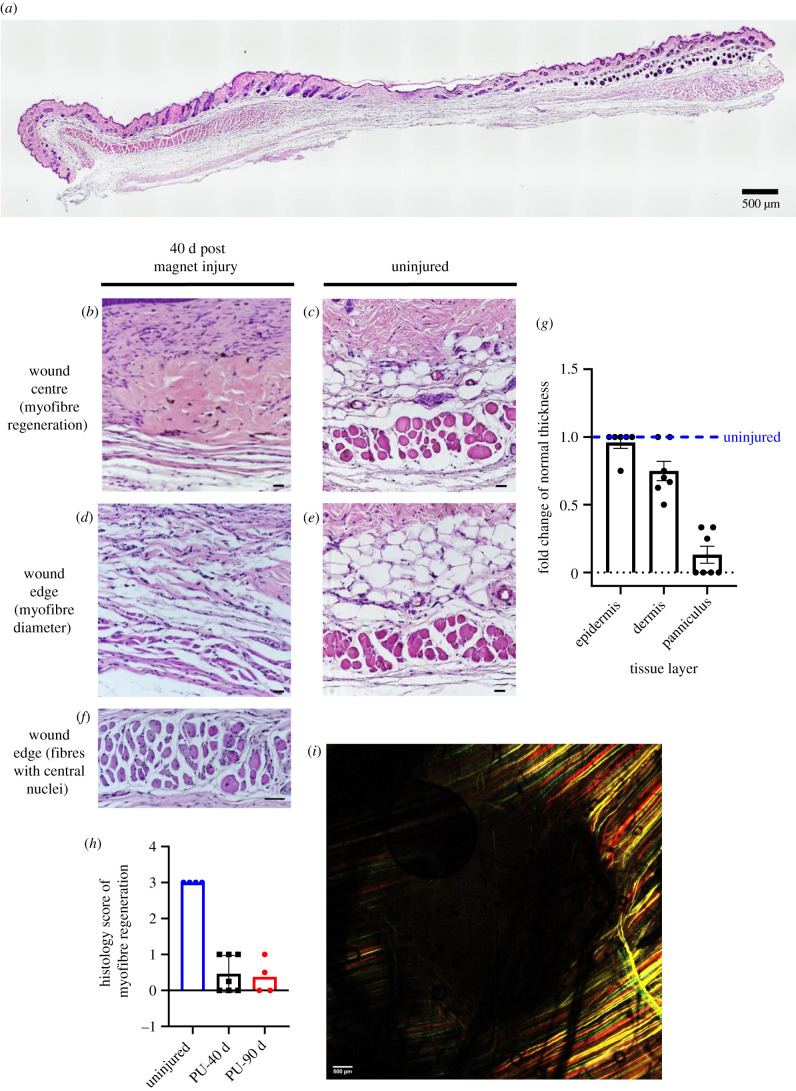
Measures and representative images of muscle regeneration between 40- and 90 d magnet-induced PU and uninjured skinfold. (*a*) A complete cross-section of wound tissue and surrounding skin at 40 d post injury, stained with H&E. Scale bar is 500 µm. (*b*–*e*) Images of the wound tissue at the centre and edge of the magnet-induced PU versus uninjured tissue. Scale bar, 50 µm. (*f*) Close-up of a wound edge (in the same mouse as (*b*)), showing fibres that have central nuclei. Scale bar, 50 µm. (*g*) Fold change in thickness of epidermal, dermal and panniculus layers, comparing pressure-injured tissues at 40 d post-PU versus uninjured control (at dashed line). *p* < 0.0001 for the difference between panniculus thicknesses. (*h*) Histological score of myofibre regeneration at 40 and 90 d post injury (PU-40 and PU-90, respectively) versus uninjured control. Note that no improvement in regeneration was observed from 40 to 90 d after injury. (*i*) A multi-channel confocal image of the wound tissue at 90 d post injury. Scale bar, 500 µm.

Forty days after magnet-induced PU, despite complete re-epithelialization, i.e. an externally healed wound (electronic supplementary material, figure S4*a*), an absence of panniculus muscle is observed in the skinfold at the wound centre, compared to the uninjured skinfold (figure 6*a*). Myofibre sizes are significantly decreased in the newly regenerated fibres of the wound edge, compared to uninjured muscle (figure 6*b*–*e*; histology score of 0.43 versus 3 respectively). Clusters of myofibres could be found that exhibited central nuclei (figure 6*f*), indicating immaturity. At 40 d, the panniculus layer had not fully regenerated, while the skin layers such as the dermis and epidermis regenerated successfully (figure 6*g*). There was no significant increase in regeneration from day 40 to day 90 post injury (figure 6*h*). The confetti fluorophores (red, green, yellow and cyan) expressed in newly regenerated myofibres can be observed under confocal microscopy. Whole-tissue confocal imaging confirmed that after 90 d, the centres of the wounded areas were devoid of fluorescence and did not contain any regenerated muscle fibres (figure 6*i*; electronic supplementary material, figure S4*b*–*d*). Circling this dark centre was newly regenerated myofibres with confetti fluorescence. These fibres were found only at the edges of the wound, not in the centre (figure 6*i*).

## 4. Discussion

We sought to understand the panniculus using three approaches: *ex vivo* cadaver studies, computational modelling and *in vivo* mouse wound healing. The purpose of microdissections in cadavers was to examine the prevalence and distribution of the panniculus in the human heel; the purpose of the computational simulations was to understand the contribution of the panniculus layer to pressure redistribution and its biomechanical protection of the soft tissues near the bony prominence, and the purpose of the mouse regeneration studies was to assess whether regeneration of the panniculus is sufficient to continue protecting the soft tissue against pressure-induced reinjury. The purpose of putting these three together is to explore the unified concept that poor panniculus regeneration might predispose human patients to PU recurrence.

In histological analyses of cadaveric human heels, a thin layer of muscle was found between the skin layers and fat pad, which varied in thickness across patients and within regions of the same patient. Prior anatomical studies of the heel have often overlooked this muscle, although some studies have identified muscle there. Cichowitz *et al*. [19] found a panniculus layer in cadaveric heels, but they suggested the panniculus to have a detrimental function in initiation of PUs, due to the intrinsic ischaemic sensitivity of muscle tissue. Specifically, muscle tissue may undergo irreversible damage due to deformation and ischaemia, thus initiating a PU. This viewpoint might appear to be the opposite of our biomechanical studies, because we suggest a protective role of the panniculus in redistributing load and decreasing regions of high deformation. However, it is possible that both viewpoints are correct: the panniculus might function to prevent PUs under normal circumstances, but under harsher conditions, for example in a vascular-disease patient or a bedridden patient, the mechanical forces might not be sufficiently redistributed to avoid PUs, in which case, ischaemia of the panniculus could initiate or worsen the PU.

In mechanical simulations of a human heel resting on a firm surface, the displacement, equivalent strain and von Mises stress were significantly increased when the panniculus muscle layer was not present. Although some tiny regions of high deformation were only mildly alleviated (figure 4*a*,*b*), the vast majority of affected regions experienced strong improvement (i.e. the vast majority of finite elements in the soft-tissue layer were cushioned from potentially injurious deformations of stress greater than 100 kPa or strain greater than 2 mm mm^−1^; figure 4*c*,*d*). In summary, when the panniculus layer is present, only a pinpoint region would be damaged. By contrast, without a panniculus layer, wide regions of soft tissue would experience cell-killing levels of stress and strain, potentially creating a significant area of tissue death.

In mouse studies, we found that the panniculus muscle failed to regenerate fully after magnet-induced PU. The margins of the wound bed contained new fibres, but stem cells and their myoblastic daughter cells did not migrate very far into the two-dimensional layer where the panniculus should be. We interpret that muscle regeneration has a limited distance of effect over a sheet-shaped defect. The regenerated muscle bore lineage labels from Pax7 + conditional fluorescence, indicating that they descended from cells that were positive for Pax7 at the time of tamoxifen induction, two weeks prior to injury. Thus, we interpret that satellite cells of the panniculus were the source of the regeneration [34]. Prior mapping of stem cell territories found that Pax7 + cells were present in the dermal territory [35] during acute skin injury, but a subsequent study [36] found those cells were not from muscle, which is consistent with our finding that labelled cells did not enter the dermis nor other non-muscle layers.

To explain why PUs tend to recur in the same location, even after a prior injury appears fully healed, we propose a novel explanation based on our combined results. After a first injury is superficially healed, the panniculus underneath may be incomplete or absent. Without the full panniculus layer, the skin and other soft tissues face increased strain and stress (in both magnitude and size of affected region) and a higher risk of tissue breakdown. Thus, the appearance of an externally healed injury may disguise the fact that important load distribution functions served by the panniculus muscle might not be repaired. Additional considerations for future clinical validation are that externally healed wounds may differ in composition, stiffness and tensile strength, compared with adjacent healthy tissues, due to incomplete regeneration of internal layers and/or due to the presence of scar tissue [37–39].

There are several caveats to this work. The heel is obviously not the only anatomical location with vulnerability to PUs, and our cadaver study did not explore the sacrum and upper trochanter. Humans have great inter-individual variability in the locations of panniculus muscle, and some people have a panniculus layer present in regions where others do not [20,40]. Furthermore, we could not conduct molecular characterization of the panniculus due to tissue breakdown. Therefore, future studies of the panniculus in the heel and other anatomical locations will be important to ascertain the nature of the panniculus, the protective role of the panniculus layer in pressure redistribution and its potential as an anatomical marker for recurrent PU prediction.

One limitation of our finite-element model is that the material parameters of the various tissue types were taken from prior publications with macroscopic mechanics. Simplified geometry fails to reflect the complexity of anatomy, much less the attributes of microarchitecture and heterogeneity, such as the non-homogeneous nature of soft tissue (adipose tissue), which includes one-dimensional structures (e.g. vessels and filaments) among the fat cells. In addition, the layers of our heel model are inseparable, and we are hence not able to study lateral movements and adhesion between the tissue layers. Finally, simulations did not compare panniculus versus scar tissue, which might occur in regions of the heel that have healed after a prior injury. Scar tissue would likely increase the risk of subsequent injury, due to its increased stiffness [39]. Although PU is affected by many influences, our results are still informative because our simulations hold these confounding variables constant while focusing on a relative comparison of the incremental effect of adding or subtracting a panniculus muscle layer from the system.

Animal models with laboratory-created pathologies can never fully recapitulate naturally arising human pathologies. Some key differences between murine and human cutaneous wound healing arise from (i) the natural tendency of murine skin to heal by contraction instead of regeneration, (ii) our mice were housed in a facility that is specific-pathogen free and infected wounds were not observed and (iii) greater HF coverage in mice, which increases the contribution of HF stem cells to cutaneous wound healing [41–43]. We attempted to minimize the HF differences by synchronizing the hair growth cycle and applying injury only to skin with HFs in telogen phase. We also believe that the impact of wound contraction is limited because we found an absence of panniculus in the externally healed skinfold, which would not have occurred if the wound had healed entirely by contraction.

Taken together, our findings show how the panniculus layer could play an important role in distributing load and providing biomechanical protection to the soft-tissue layers around it, thus questioning the widely held notion that the panniculus is vestigial and holds no functional significance in humans. Pressure-induced muscle damage led to incomplete regeneration of the panniculus muscle layer, which could deprive the surrounding soft tissues of mechanical protection and promote recurrence of PUs. There is indeed a layer of panniculus in the human heel, and more research should be done to study the predictive value of an intact panniculus after the PU has healed, towards the prediction of PU recurrence and towards the prioritization of clinical resources to prevent recurrence.

## Data accessibility

All modelling files are available to the public free-of-charge at https://github.com/ComputationalBioLab/panniculus_simulations.
